# Development of an integrated Monte Carlo model for glioblastoma multiforme treated with boron neutron capture therapy

**DOI:** 10.1038/s41598-017-07302-9

**Published:** 2017-08-01

**Authors:** Leyla Moghaddasi, Eva Bezak

**Affiliations:** 1 School of Physical Sciences, University of Adelaide, Adelaide, Australia; 2 Department of Medical Physics, Adelaide Radiotherapy Centre, Adelaide, Australia; 3 School of Health Sciences, University of South Australia, Adelaide, Australia; 4 Sansom Institute for Health Research, University of South Australia, Adelaide, Australia

## Abstract

Glioblastomas (GBM) are notorious for their high fatality rate. Boron Neutron Capture Therapy (BNCT) being a biochemically targeted type of radiotherapy is a potent modality for GBM. In the current work, a BNCT treatment modelling framework for GBM was developed. Optimal Clinical Target Volume (CTV) margins for GBM-BNCT and the BNCT efficacy have been investigated. The model integrated a cell-based dosimetry model, an in-house-developed epithermal neutron beam model and previously-developed Microscopic Extension Probability (MEP) model. The system was defined as a cubic ICRP-brain phantom divided into 20 *μ*m side voxels. The corresponding ^10^B concentrations in GBM and normal brain cells were applied. The *in-silico* model was irradiated with the epithermal neutron beam using 2 and 2.5 cm CTV margins. Results from the cell-based dosimetry and the MEP models were combined to calculate GBM cell survival fractions (SF) post BNCT and compared to x-ray radiotherapy (XRT) SFs. Compared to XRT, the SF within the beam decreased by five orders of magnitudes and the total SF was reduced three times following BNCT. CTV extension by 0.5 cm reduced the SF by additional (53.8 ± 0.3)%. In conclusion, BNCT results in a more efficient cell kill. The extension of the CTV margin, however, may not increase the treatment outcome significantly.

## Introduction

Glioblastoma Multiforme (GBM) is the most malignant astrocytic glioma (grade 4)^1^ and is known for its aggressive proliferation and extensive invasion to normal tissue before any symptoms are presented. Biochemical features allow the tumour cells to invade extracellular matrix of the normal brain^2^. GBMs present clinically with extensive hypoxia (both transient and acute), high intrinsic radioresistance with genetic heterogeneity and complexity. GBMs are associated with very poor prognosis and currently there is no curative treatment for this malignancy. Despite aggressive treatment regimens, including concomitant and adjuvant temozolomide and x-ray therapy, less than 27% of patients are expected to survive more than two years^3^.

The exact incidence and entire extent of tumour microscopic spread, to be encompassed by CTV, cannot be visualised by any current imaging techniques^4^. As a result, determination of an optimal radiotherapy Clinical Target Volume (CTV) is primarily empirical and hence subject to uncertainty. The infiltrative growth pattern of GBM and its rapid peripheral expansion cause the CTV delineation of this tumour to be even more problematic. In addition, histopathological studies on the extent of GBM cell infiltration into normal tissue, beyond the Gross Tumour Volume (GTV), show large discrepancies^5^. Marginal and distant GBM recurrences reported in literature^6, 7^,could be attributed to the Microscopic Extension (ME) uncertainty, while the high rate of local relapse^8–10^, could be explained by intrinsic radioresistance of GBM stem cells and their other aggressive features.

### Alternative treatment modalities - Boron Neutron Capture Therapy

Developments in x-ray External Beam Radiation Therapy (EBRT) in terms of increased precision of dose delivery over the last two decades have failed to offer markedly improved outcomes for GBM patients. As a result, there is need for other treatment modalities to be investigated and optimised for high grade gliomas. Boron Neutron Capture Therapy (BNCT) is a biochemically-targeted type of radiotherapy based on nuclear capture reactions which occur when a thermal neutron is captured by a stable ^10^B atom, resulting in emission of an energetic alpha particle (∼1.47 → 1.78 MeV), a recoiling^7^Li nucleus (∼831.6 keV → 1.01 MeV) and a 478 keV gamma ray, see equation (1) for a list of possible reactions. The alpha particles and^7^Li nuclei deposit their energy along the range of approximately 4 to 7 *μ*m with a high Linear Energy Transfer (LET) of ∼240 keV/*μm* ^11, 12^. BNCT is a binary modality in which a suitable ^10^B agent is taken up preferentially by malignant cells following administration of a boron agent.

1 $$\begin{array}{c}{}^{1}n+{}^{10}B\to {}^{11}B+\gamma \\ {}^{1}n+{}^{10}B\to {}^{7}Li+{}^{4}He+\gamma \\ {}^{1}n+{}^{10}B\to {}^{7}Li+{}^{4}He\end{array}$$

Clinical trials of BNCT for malignant gliomas were undertaken at Brookhaven National Laboratory (BNL) and Massachusetts Institute of Technology (MIT) between 1959 and 1961^13^. This was followed by a large pause in further research due to a failure to show considerable improvement in survival times^13^. Following the technological advances that enabled production of epithermal neutron beams^14^ and development of more suitable boron agents containing ^10^B, BNCT studies re-emerged in the 1990s^15^. Subsequently, encouraging results in terms of lifespan improvement of GBM patients post BNCT were reported. Yamamoto *et al*.^16^ reported a 2-years survival rate of 53.3% for a series of 15 GBM patients which was superior to that observed in RTOG trial using EBRT (35%)^17^. Kawabata *et al*.^18^ investigated the survival benefit of BNCT by comparing survival times of 21 patients treated with BNCT against 27 patients treated with x-ray EBRT. Improved survival times, from 48.2 to 76.2 % and 14.8 to 25.0 % for 1-year and 2-years survival, respectively, were observed for patients treated with BNCT. Survival benefit of BNCT for recurrent malignant gliomas following surgery and conventional EBRT has also been reported in the literature. Median survival time for recurrent GBM patients treated by BNCT was found 8.7 months (n = 12), 7.5 months (n = 7), and 9.6 months (n = 19) in a Swedish^19^, Finnish^20^, and Japanese studies^21^, respectively. The median survival time for recurrent GBM patients was found 5.8 months (n = 225) in phase II chemotherapy trials^22^. However, these results should be viewed with caution as the patient numbers were small and the comparisons were not randomized. In addition, not all patients included in the above-mentioned studies (high grade gliomas) had GBM histopathology confirmed, thus possibly exaggerating the GBM survival rates.

The BNCT modality has several physical and biological advantages over megavoltage x-rays for the radiotherapy of GBM with its inherent complexities:

1. The treatment is delivered by high LET particles. The clustered damage, in form of DNA double strand and single strand breaks, produced by high LET radiation beam makes the repair of sublethal and potentially lethal damages less efficient. This increases the possibility to overcome GBM s intrinsic radioresistance and aggressive features and offers a higher tumour kill/sterilisation compared to x-ray radiotherapy.
2. As BNCT is a binary modality, it has the potential to selectively deliver localized dose to tumour cells (containing substantially higher concentrations of ^10^B compound) dispersed in normal tissue, while minimizing normal tissue toxicity. This makes BNCT an attractive modality for GBM which grows infiltratively rather than forming a solid tumour^23^.
3. Charged particulate radiation interacts with biological tissue predominantly through direct interaction of incident particles or secondary electrons from ionized atoms with DNA, rather than producing free radicals which in return cause DNA damage. As a result, the cell killing process in BNCT is less sensitive to oxygen pressure compared to x-ray radiotherapy^24^, which makes it advantageous for tumours with extensive hypoxia such as GBM^25^.

Although BNCT theoretically appears very suitable for GBM and other tumours with aggressive features, the success of this technique depends on three important factors: a sufficient amount of ^10^B in tumour cells (at least 20 *μ*g/g or 10^9^ atoms/cell^15^), differential uptake of boron in tumour and normal cells and sufficient fluence of thermal neutrons delivered to the site of the tumour.

#### Boron concentration

The two commonly used boron delivery agents are: sodium mercaptoundecahydro-*closo*-dodecaborate (*Na*_2_ *B*_12_ *H*_11_ *SH*) or commonly named BSH; and (L)-4-dihydroxy-borylphenylalanine or BPA^15^. Boron concentrations in normal brain and glioma cells using these agents have been investigated in several studies. In an animal study, Barth *et al*.^26^ reported a ^10^B concentration of 11.8 ± 3.5 and 94.5 ± 69.1 *μ*g/g in normal brain and tumour cells respectively, using BPA with induced Blood-Brain Barrier Disruption (BBB-D), and 4.4 ± 1.8 and 48.6 ± 17.2 *μ*g/g in normal brain and tumour cells respectively, using BSH with BBB-D. As shown in Table 1, other studies suggest a smaller ratio of tumour to normal tissue ^10^B concentrations^20, 27–31^. Capuani *et al*.^32^ investigated the effect of preloading L-DOPA prior to BPA infusion and concluded that L-DOPA had a potential use as BPA accumulation enhancer, increasing tumour ^10^B concentration from 33.5 ± 7.5 *μ*g/g without preloading to 88.3 ± 12.1 *μ*g/g with preloading.

**Table 1:** A summary of ^10^B concentrations in normal brain (NB)/blood and tumour cells.

Author	Dose (mg/Kg)	^10^B compound	^10^B concentration (*μ*g/g)	
			Blood/NB	Tumour cell
Barth^26^	500	BPA	11.8 ± 3.5	94.5 ± 69.1
Barth^26^	65	BSH	4.4 ± 1.8	48.6 ± 17.2
Capala^27^	900	BPA	15–34	3.7 × NB
Joensuu^20^	290	BPA	12–15	3.5 × NB
Diaz^28^	250–330	BPA		3.5 × NB
Coderre^29^	250–330	BPA	12–16	3.5 × NB
Palmer^30^	250–350	BPA	10–15	3.5 × NB

#### Neutron spectrum for boron neutron capture therapy

An optimal neutron spectrum for BNCT should contain high epithermal (0.25 *eV*–100 *keV*) and minimal fast neutron (E > 100 *keV*) components^30^. The cross section for the ^10^B(n,*α*)^7^Li neutron capture reaction is high in the thermal energy range of neutrons (E < 0.25 *eV*). However, the penetration depth for thermal neutrons is limited. Consequently, epithermal neutrons are more useful for clinical purposes as they lose energy while traversing the medium and will undergo boron neutron capture at larger depths.

### Monte Carlo particle simulations

Bio-mathematical and computational modelling is appreciated as a useful tool to provide predictions of the probable response of a tumour to radiotherapy for a variety of circumstances, including different therapeutic regimens and treatment modalities.

Geant4^33^ is a flexible and powerful Monte Carlo (MC) toolkit capable of simulating complex experimental set-ups. This MC toolkit, which is the result of an ongoing worldwide collaboration, has been developed to precisely track the passage of radiation through matter for a diverse range of particles and interaction processes over an extended energy range. The diverse functionality, inclusion of hadronic processes and performance optimization features make Geant4 a suitable MC software/tool for BNCT simulation. Nevertheless, the use of Geant4 for simulation of a BNCT radiation treatment framework has been limited to our knowledge.

Previously, a comprehensive and flexible GBM x-ray radiation therapy modelling framework was developed by our group^34^. Using Geant4 and MATLAB^®^ (Mathworks, Natick, MA), GBM and its surroundings, containing both normal and clonogenic cells, was modelled. The model was used to evaluate current CTV practices applied in x-ray EBRT^34^, in terms of GBM cell survival following a treatment with a 6 MV photon beam for several types of microscopic extension distributions. The model was further developed to incorporate GBM heterogeneous radiosensitivity and hypoxia^35^.

The purpose of the current work was to significantly extend the GBM model to incorporate BNCT and to investigate the effect of CTV margins as well as BNCT efficacy as compared to x-ray EBRT in terms of GBM cell survival.

The following section summarises the structure of the GBM treatment model designed previously.

### Review of the microscopic scale GBM treatment model

**Figure 1:**
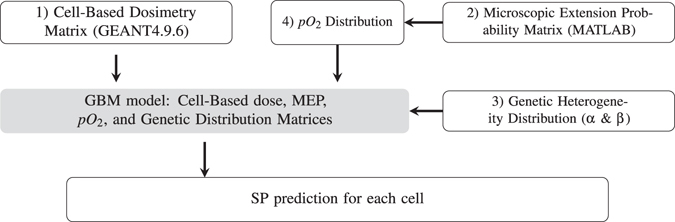
diagrammatic representation of the structure of the model components developed in the previous work^34^.

The comprehensive GBM treatment modelling framework developed consists of a microscopic extension GBM model, a tumour irradiation model, and a Survival Fraction (SF) algorithm^34, 35^, as shown in Fig. 1.

The cell-based dosimetry module has been developed using Geant4 (version 9.6.p01) particle tracking toolkit^33^ to calculate the absorbed dose in each cell of a spatial brain glioblastoma model. The geometry scale has been chosen to cover the entire GBM microscopic extension in all directions. To enable cellular based dosimetry, the simulated volume has been divided into 20 × 20 × 20 *μm* ^3^ cubic voxels (the average size of glioma cells^36^).

**Figure 2:**
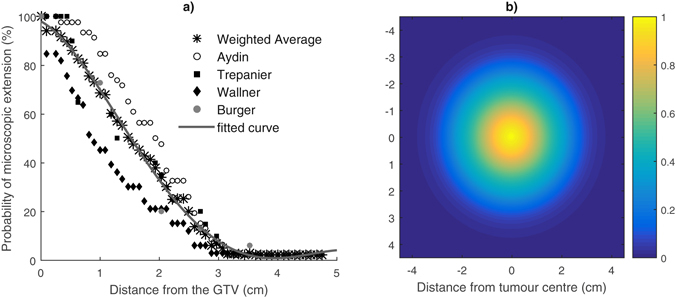
(**a**) Clinical studies investigating the extent of microscopic extension in GBM patients. The function fitted to combined results (a three-term Gaussian function) was considered as the basis for the MEP models; (**b**) Isotropic circular MEP distribution; courtesy of Moghaddasi *et al*.^35^.

The invasion of tumour cells within the normal brain tissue (i.e. the Microscopic Extension Probability (MEP) model), has been modelled in MATLAB^®^ using a probability distribution function. The function assigned each cell with a probability of being a tumour cell as a function of distance from the GTV. This probability distribution function was obtained from published data reporting on GBM recurrence patterns^6, 37–39^, see Fig. 2 ^35^. The function fitted to the combined data, equation (2) forms the basis of the MEP model used in this work:

2 $$ME{P}_{ij}=(\begin{array}{cc}{a}_{1}\exp (-{(\tfrac{{x}_{i}-{b}_{1}}{{c}_{1}})}^{2})+{a}_{2}\exp (-{(\tfrac{{x}_{i}-{b}_{2}}{{c}_{2}})}^{2})+{a}_{3}\,\exp \,(-{(\tfrac{{x}_{i}-{b}_{3}}{{c}_{3}})}^{2}) & {\rm{for}}\,{x}_{i}\le {\rm{4}}\mathrm{.1}(\mathrm{cm})\\ 0 & {\rm{for}}\,x > 4\mathrm{.1}(\mathrm{cm})\end{array}$$

where *x*_*i*_ represents the radial distance of the voxel *ij* from the GTV surface. An isotropic circular MEP has been modelled as this assumption is made in clinics for delineation of CTV margins. For this pattern, both the tumour and its ME have been considered circular with the MEP reducing isotropically in all directions based on the function above.

In the final simulation step, the dose matrices calculated in Geant4 are exported into MATLAB^®^ and are combined with the MEP models to obtain cell-based survival fractions. The GBM model can incorporate other biological characteristics of GBM cells including heterogeneous radiosensitivity and hypoxia^35^. Individual survival probabilities (SP) of all cells are calculated using Linear Quadratic (LQ) theory, and are combined to calculate SFs for any specific region (e.g. within the beam, and penumbra).

## Results

### Geant4 cell-based dosimetry for BNCT treatment

**Figure 3:**
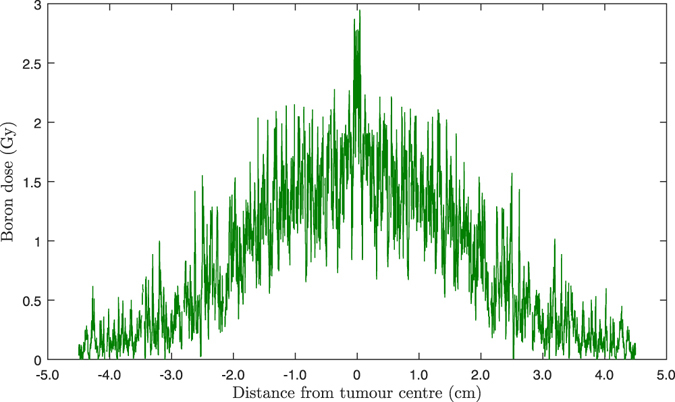
Transverse profile of the calculated cell-based boron dose distribution (combined ^7^Li and alpha dose matrices) in the scoring plane versus distance from the tumour centre. The statistical uncertainty of the calculated results (the relative error between several runs) was ∼±12.5%.

Figure 3 shows the transverse profile of the calculated boron dose (the combined ^7^Li and alpha dose matrices). It is evident that the boron dose reaches maximum within the GTV region (1 mm radius) and reduces at larger distances from the GTV as a result of declining boron concentration in cells.

**Figure 4:**
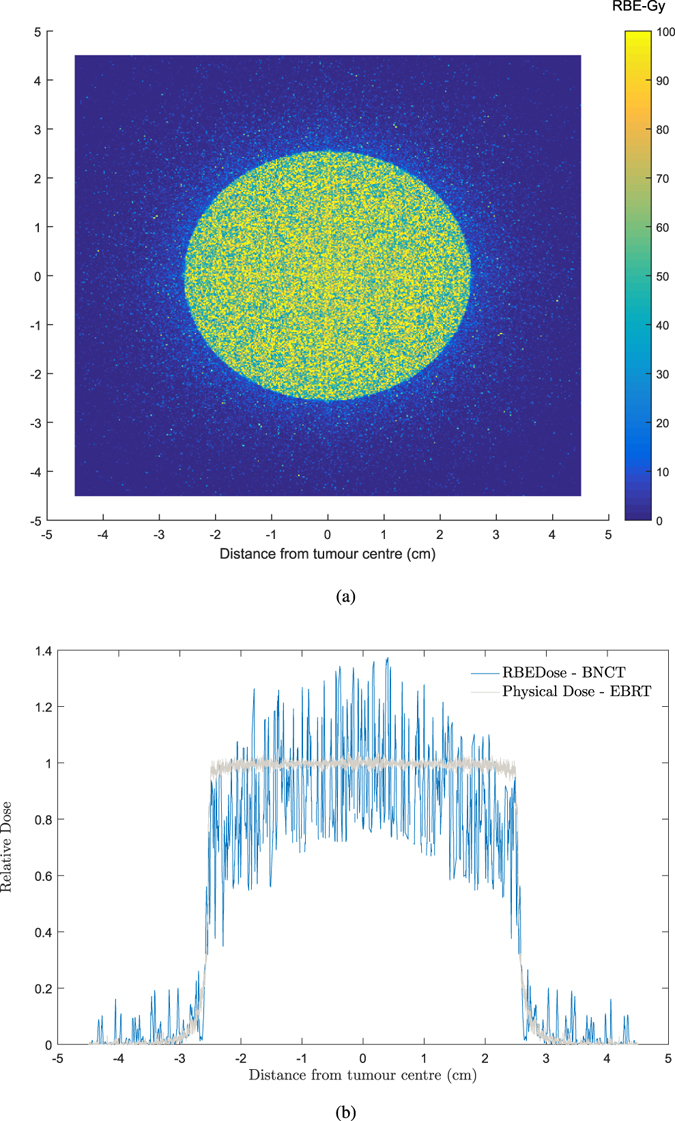
Cell-based RBE dose using equation (4) and four Geant4-calculated cellular dose matrices. (**a**) RBE dose distribution in the scoring plane versus distance from the tumour centre. (**b**) Transverse RBE dose in the scoring plane versus distance from tumour centre. The transverse profile for x-ray EBRT^34^ is also shown as a comparison. The statistical uncertainty of the calculated results (the relative error between several runs) was ∼±10%.

The distribution of the total RBE dose matrix in the scoring plane is shown in the Fig. 4a. It shows that there is considerable scattered neutron dose beyond the PTV.

The transverse profile of the total RBE dose in the scoring plane is presented in Fig. 4b. As expected, the transverse profile for BNCT falls rapidly in the PTV region, which is in contrast to x-ray beam transverse profiles, that are reasonably flat in the PTV region.

### Assessment of survival fractions for 2.0 cm clinical target volume margin

The results in this section are presented in terms of (a) SF within the beam (the PTV), (b) SF within the penumbra region (5.0 mm beyond the PTV), (c) total SF (including out of beam (<1%), within the beam and penumbra region) utilizing circular MEP model for a GBM consisting of homogeneous population of GBM cells. The results for conventional x-ray therapy are also presented using the previously-developed model^34^ for comparison purposes.

Table 2 summarizes SF values in the regions of interest as well as the total SFs. The SF within the beam has reduced by about four orders of magnitude for BNCT modality compared to SF following a conventional x-ray therapy. While not at the same magnitude, the SF in penumbra region also showed a marked improvement using BNCT as compared to x-ray therapy.

**Table 2:** Survival fractions (SFs) in different regions for homogeneous-hypoxic circular MEP model for BNCT and conventional photon modalities.

	BNCT modality	X-ray conventional modality
SF within the beam (%)	0–10^−6^	(3.22 ± 0.18) × 10^−3^
SF within the penumbra region (%)	2.74 ± 0.49	6.71 ± 0.88
Total SF (%)	3.92 ± 0.68	12.79 ± 0.97

The results indicate a reduction in the total SF by a factor of 3 between BNCT and conventional photon therapy. It is worth noting that for the circular MEP model, the total number of tumour clonogens and the number of tumour clonogens in the penumbra region before the treatment were 2,908,500 and 299,420 respectively.

**Figure 5:**
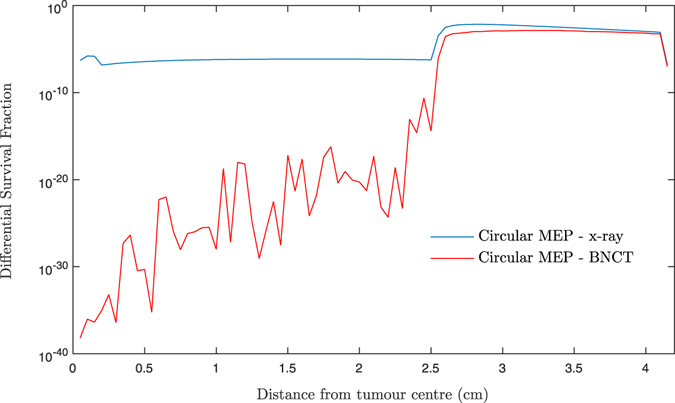
Differentiated SF versus distance from tumour centre for two scenarios: homogeneous-hypoxic GBM models treated by (1) BNCT; and (2) conventional x-ray therapy. These graphs are obtained applying “circular” MEP model and 2.0 cm CTV (2.5 cm beam radius).

Figure 5 shows the calculated differential SF curves in 0.5 mm steps. The results for treatment of homogeneous hypoxic GBM treated with BNCT and conventional x-ray therapy are presented to allow comparison and analysis of the SF pattern at any distance. Considering the logarithmic scale on the vertical axis (differential SF), a marked decrease in the SF within the beam was observed for BNCT as compared to x-ray modality. The SF within the beam for BNCT treatment gradually increased with distance from the tumour centre prior to a rise in the penumbra region.

Nevertheless, the SF remained almost the same throughout the beam area for the GBM model treated with x-rays. This is attributed to the dose gradient within the beam for BNCT treatment, see Fig. 4b, as a result of declining ^10^B content as a function of distance from the GTV.

### Quantification of survival fraction reduction following a CTV margin extension

**Figure 6:**
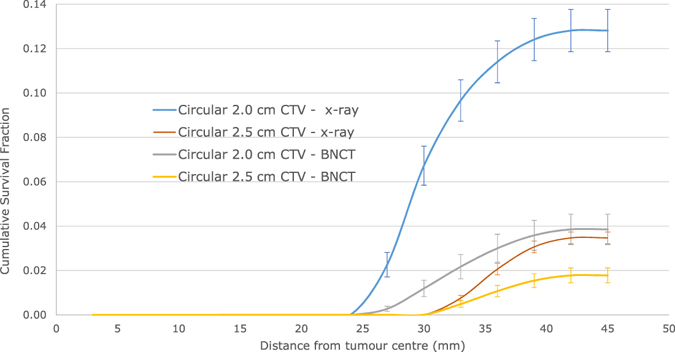
Cumulative SF versus distance from tumour centre for 2.0 and 2.5 cm CTVs applying the circular microscopic extension distribution model for a homogeneous-hypoxic GBM treated by BNCT as compared to conventional x-ray therapy.

Cumulative survival fractions corresponding to a 2.0 cm CTV margin (5.0 cm PTV) and 2.5 cm CTV margin (6.0 cm PTV) for the circular MEP model and for a genetically homogeneous-hypoxic GBM model are shown in Fig. 6 for BNCT as compared to XRT. As illustrated, compared to x-ray radiotherapy, BNCT results in significantly lower SFs for both 2.0 and 2.5 cm radii beams.

Calculated *SF*_*change*_ data, as a result of the CTV increase by 0.5 cm, are summarized in Table 3. The SF was reduced by 53.83 ± 0.31% following BNCT. The reduction in SF as a result of the CTV extension (by 72.93 ± 0.06%) was more pronounced in case of conventional x-ray therapy.

**Table 3:** Changes in SFs as a result of the CTV extension (from 2.0 to 2.5 cm).

Treatment modality	SF- 2.0 cm CTV (%)	SF- 2.5 cm CTV (%)	*SF*_*change*_(%)
BNCT	3.85 ± 0.68	1.78 ± 0.33	53.83 ± 0.31
Conventional x-ray therapy	12.81 ± 0.95	3.47 ± 0.26	72.93 ± 0.06

## Discussion

In the previous report an integrated MC quantification tool was developed for evaluation of survival fraction of a GBM tumour, depending on its clonogenic microscopic spread (MEP distributions)^34, 35^, and different CTV margin extensions (2.0 and 2.5 cm), following an x-ray EBRT. The establishment of a cell-based simulation platform allowed for application of different radiation sources/modalities and of different radiobiological and physical (e.g. cellular material composition) cell properties. In the current work, the GBM model was utilized to develop a BNCT GBM treatment modelling platform for a homogeneous population of GBM cells.

The rationale for BNCT is its ability to deliver a localized high LET absorbed dose to tumour cells (containing a sufficient number of ^10^B atoms), while minimizing the dose to normal tissue cells which contain a fewer number of boron atoms. Therefore, an effective and nontoxic BNCT depends on the selective uptake of a boron compound by tumour cells or, in other words, on the ratio of tumour to normal tissue boron concentration. It is known, however, that glioblastoma multiforme tumours present with low uptake due to their necrotic and hypoxic nature^40^ as compared to other tumours (e.g. melanoma). In our study, a ratio of 3.5 was considered as this is the most commonly assumed value in literature (Table 1). Ongoing research is addressing this issue by investigating how the tumour uptake can be enhanced. An encouraging tumour to blood boron concentration ratio of 8.3 ± 0.7 was obtained using L-DOPA administration prior treatment^32^.

The calculated boron dose showed a peak within the GTV region, followed by a sharp gradient reduction, reaching ∼30% of its maximum at 2.5 cm from the tumour centre (Fig. 3) and resulting in an bell-shaped dose curve within the beam region (which is normally flat for conventional EBRT) as shown on the transverse profile of total RBE dose (Fig. 4b). A beam with such a profile allows to escalate the dose within the GTV as the absorbed dose reduces at larger distances due to reduced boron concentration which in turn depends on the probability that the cell is a tumour cell.

The calculated SF, following BNCT, within the beam region decreased by three orders of magnitude as compared to conventional x-ray EBRT for homogeneous hypoxic GBM (Table 2). The calculated number of surviving cells within the beam (*SF*_*within the beam*_ × *total number of tumour cells before treatment*) was ∼0.01 which suggest that BNCT should, in principle, be able to provide local tumour control for GBM. Considering that in the current work a simplified model of GBM with homogeneous radiation sensitivity and isotropic infiltration distribution has been simulated, this finding should be considered with care. Nevertheless, published clinical reports, while scarce, are supportive of this result. Miyatake^41^ presented a case report of the recurrence pattern of a gliosarcoma patient after BNCT and it was demonstrated that gliosarcoma responded well to BNCT and the recurrence occurred in the periphery of the tumour outside the treatment field. Likewise, evidence of favourable local tumour control of high-grade meningiomas using BNCT was demonstrated in a cohort of 20 patients studied by Kawabata^42^. Only three out of twenty patients had local treatment failure^42^. It should be noted though that high-grade meningiomas show a slightly higher ratio of tumour to normal brain boron concentration (3.7 ± 0.8)^42^ compared with malignant gliomas and as a result the local control for GBM should be slightly inferior to that of meningiomas. Matsuda^43^ analysed the failure pattern of 8 GBM patients treated by BNCT. None of the patients recurred within the GTV and 6 out of 8 patients showed failure in the low dose region of CTV (i.e. GTV + 2 cm). The low dose regions were a result of low boron concentrations and an insufficient delivery of thermal neutrons. An improved local control for GBM should be expected if these two factors can be overcome.

Survival fractions in the penumbra region were also investigated to quantify the contribution of local invasion/microscopic disease, which could be eliminated by selecting a slightly larger CTV margin, to total SF. The SF in penumbra for BNCT was lower than that for x-ray EBRT by approximately 2.5 times. This is likely a result of the boron dose produced by neutrons which have thermalised through lateral scattering and being captured within the penumbra region The total SF was also significantly lower (by ∼3 times) for BNCT as compared to photon therapy.

The differentiated curves presented in Fig. 5 allowed analysis of SF at any distance from the GTV. Given the logarithmic scale on the vertical axis, it can be observed that SF within the beam is significantly lower following BNCT as compared to x-ray therapy. Unlike the x-ray EBRT, the SF for BNCT gradually increases as a function of distance from the tumour centre as a result of boron dose reduction. This trend can explain the observed failure pattern in the study of Matsuda^43^ where 6 out of 8 patients recurred within CTV and none had failed within the GTV. As widely reflected in the literature^9, 37, 44^, tumour relapse for x-ray therapy shows a different trend, with GBM recurring locally and particularly in the GTV region containing cells with severe hypoxia. It is interesting to note that this clinical observation is evident in the current work’s results. The SF for photon therapy showed a local peak within the GTV (1.0 mm radius), Fig. 5, despite a 10 Gy boost dose delivered to this region, suggesting that the tumour most likely relapse in the GTV or beam region. The SF in penumbra, while lower for BNCT, is high for both treatment modalities which increases the risk of preferential recurrence, as also demonstrated in published clinical studies^42, 41^.

It is clearly evident that increasing the beam size will reduce the SF, and of course, simultaneously increase normal tissue toxicity. However, a quantification of the reduction of SF when the CTV is extended could be a valuable guidance in adjusting dose prescriptions. In order to investigate the impact of the extent of the CTV margin, the beam size was increased by 0.5 cm in radius (resulting in 2.5 cm CTV margin), and cumulative SFs were calculated and plotted vs distance from the tumour centre. As indicated in Table 3 and shown in Fig. 6, total SFs both for 2.0 and 2.5 cm CTV margins were markedly lower using BNCT as compared to conventional x-ray therapy. However, the change in the SF as a result of CTV extension by 0.5 cm was not as pronounced for BNCT, as compared to CTV in extension in case of x-ray EBRT (Table 3). In other words, while the CTV extension could be beneficial for GBM patients treated by x-ray EBRT, it does not seem to be as advantageous for patients treated by BNCT, particularly considering the toxicity induced to normal tissue by scattered neutron having a much higher detrimental effect than photons.

In conclusion, an integrated radiobiological framework has been developed using the Geant4 Monte Carlo toolkit and MATLAB to evaluate current CTV margin extensions in terms of cell kill efficacy for glioblastoma of the brain using BNCT. The efficacy of BNCT has also been investigated as compared to x-ray EBRT.

The main outcomes of this study are: (1) according to these simulations, while radiation-induced damage caused by x-ray beams may not be sufficient to kill or sterilise GBM cell populations and the tumour is most probably to relapse in the treatment volume, BNCT could provide local control for GBM patients; (2) A quantification tool has been developed to estimate the reduction in the tumour cell survival fraction due to extension of the CTV. Although the SF is sensitive to the CTV margin extension in BNCT, this reduction may not justify the radiation injury to healthy brain cells caused by high LET BNCT beam in its current state of technological development. The efficacy of BNCT strongly depends on the number of low energy neutrons in a spectrum as well as on a high ratio of tumour to normal brain boron concentrations. Should these factors be optimized, BNCT could be a potent modality for GBM.

We acknowledge that this study has utilized a simplified model of GBM and its microscopic extension. It is a general knowledge that cancer systems, particularly GBM, are complex and dynamic systems where factors such as the micro-environmental changes, immune system response, and cellular phenotype conversions affect progression through carcinogenesis. Another limitation of this model is the application of modified LQ model to translate the physical dose to radiobiological endpoint (e.g. cell kill). Tumour cell kill is a complicated procedure, particularly for GBM with its inherent complexities, and is influenced by a large number of microscopic biological and chemical mechanisms which are not fully incorporated in the LQ model^45^. From a mathematical perspective and irrespective of radiobiological reasons, the validity of the LQ model (based on Poisson-distributed lesion assumption) diminishes at large doses and the cell survival is underestimated by the model^46, 47^. In other words, the model developed in this work may overestimate cell kill efficacy using BNCT.

The GBM model in this work consisted of a population of cells with homogeneous radio-sensitivity and an isotropic infiltration distribution (circular MEP). In future, genetic heterogeneity of GBM will be incorporated in the BNCT treatment modelling and anisotropic infiltration distributions will be investigated. Another important area of future development of the present work is to use more accurate models, utilizing particle track structure parameters, to translate physical dose to radiobiological effect.

## Methods

The modelling framework described above has been used as the basis for the GBM-BNCT model development. In case of BNCT, the cell-based dosimetry module, however, is not independent of the GBM MEP model as the dose deposited in individual cells depends on the cellular boron concentration which itself is dependent on whether the cell is a tumour or a normal cell. These components are discussed in the following sections.

### Cell-based dosimetry for BNCT

#### Geometry design

**Figure 7:**
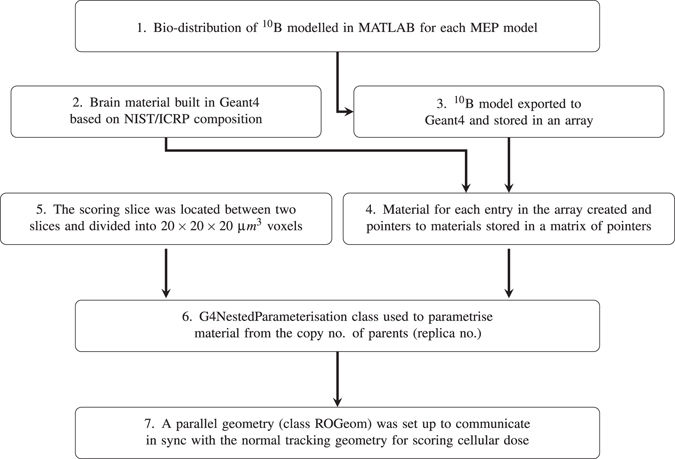
Schematic diagram showing the architecture of the geometry of Geant4 cell-based dosimetry system in the current work.

**Figure 8:**
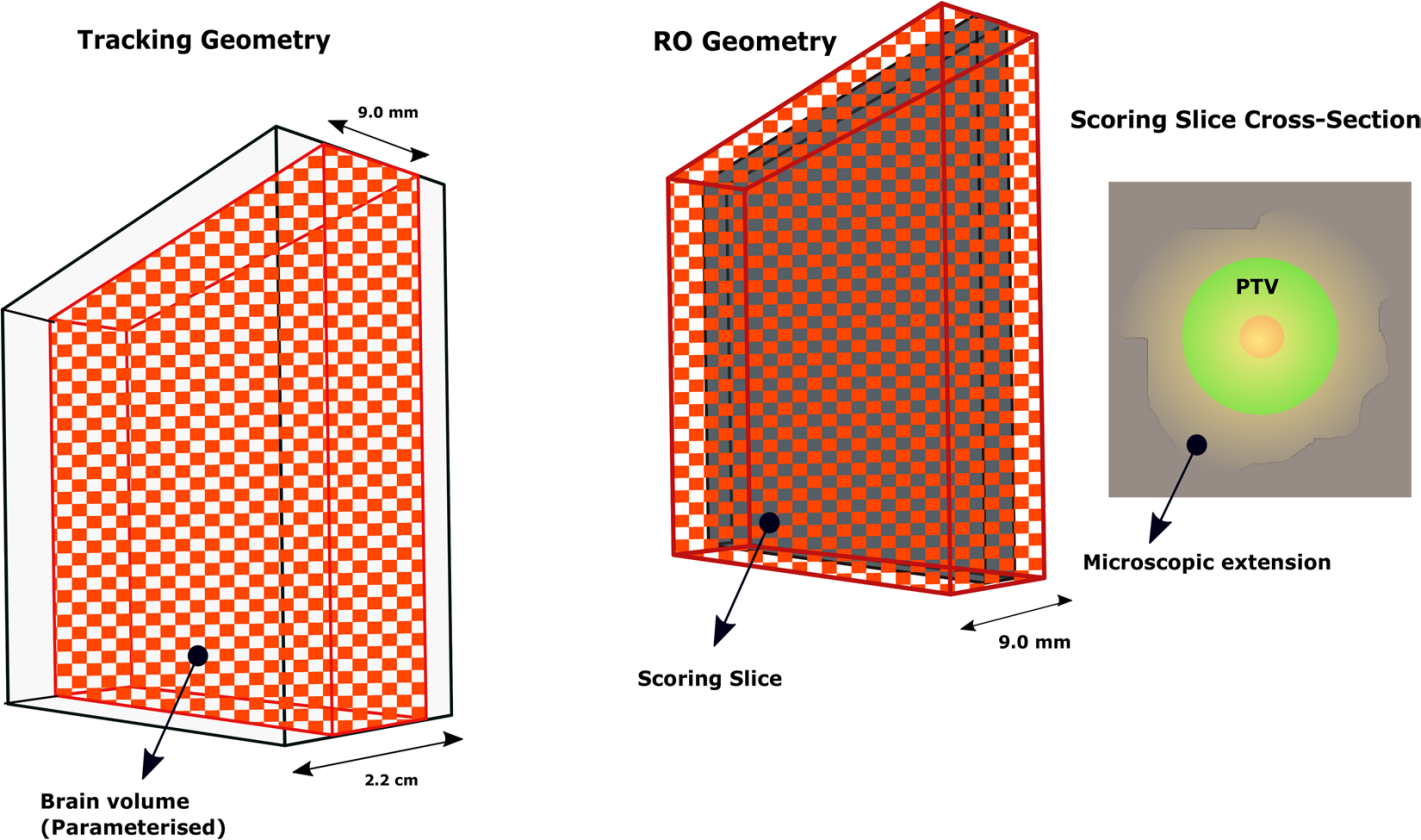
The real tracking geometry is shown on the left. A 9 *cm* × 9 *cm* × 2.2 *cm* box enclosed the brain volume (9 *cm* × 9 *cm* × 9 *mm*) which was divided into 3 × 3 × 3 *mm* ^3^ and the material parametrisation was implemented for all voxels within. In the middle, the RO geometry is shown which consisted of the brain volume enclosing the scoring plane located in the middle of the volume and divided into 20 × 20 × 20 *μm* ^3^ (the size of glioma cells). The RO geometry communicates in sync with tracking geometry to score the dose in the cells defined in the scoring plane. On the right, the cross section of the scoring plane is shown. It should be noted that this diagram is not to scale to allow illustration of details.

The architecture of the geometry used for cell-based dosimetry is shown in Fig. 7. The simulated geometry was a 9 *cm* × 9 *cm* × 2.2 *cm* brain phantom consisting of a spherical GTV (1 mm diameter) and a microscopic extension region (spanning up to 4.1 cm from the GTV) and a 0.4 cm Planning Target Volume (PTV). The size of the GTV was intentionally considered small as the main aim of this study is to investigate the effect of the CTV margin extension on the SF and the larger size of the GTV only increases CPU time. This enclosed a dose scoring volume of 9 *cm* × 9 *cm* × 0.9 *cm* (the volume in checked print in Fig. 8). This volume was divided into 20 × 20 × 20 *μm* ^3^ and each cell within this volume was assigned its corresponding ^10^ *B* concentration in addition to the brain material. While the Geant4 particle tracking was performed in the entire geometry, the dose was scored in a single slice with 9 *cm* × 9 *cm* × 20 *μm* dimensions located at 2.0 cm below the phantom surface, perpendicular to the beam direction.

#### Bio–distribution of boron in brain

In order to simulate the brain infused by a ^10^B agent in Geant4, each cell was assigned a brain material and a boron concentration (see steps 2 and 3 in Fig. 7). The required fields to specify and construct the brain material (name, density, and atomic constituents) in Geant4 were taken from the National Institute of Standards and Technology (NIST) Listings, “Compositions of Materials used in STAR Databases” webpage: http://physics.nist.gov/cgi-bin/Star/compos.pl?matno = 123 (see Table 4).

**Table 4:** Atomic constituents of brain material taken from National Institute of Standards and Technology (NIST) Listings, “Compositions of Materials used in STAR Databases” webpage.

Symbol	Fraction Mass	Symbol	Fraction Mass
H	0.110667	S	0.001770
C	0.125420	Cl	0.002360
N	0.013280	K	0.003100
O	0.737723	Ca	0.000090
Na	0.001840	Fe	0.000050
Mg	0.000150	Zn	0.000010
P	0.003540		

source by: http://physics.nist.gov/cgi-bin/Star/compos.pl?matno=123.

Based on the data summarized in Table 1, boron concentrations of 13 and 45.5 *μg*/*g* were assumed for normal brain and GBM cells (i.e. normal brain boron concentration × 3.5), respectively. Using these concentrations a linear function was derived to determine cellular boron concentration as a function of the probability that the cell is a tumour clonogen (MEP) (equation (3)). This assumption is justified based on clinical observations indicating that there is a linear relation between blood and tumour boron concentrations:^27^

3 $$B\_concentratio{n}_{ij}=32.5\times ME{P}_{ij}+13$$

where *B*_*concentration*_*ij*_ is the boron concentration in the cell (voxel) ij with the *MEP*_*ij*_ probability that the cell is a tumour cell. The linear function was then converted to a step function with twelve steps. Such derived bio-distribution of boron (the step #1 in the flowchart 1) was exported from Matlab to Geant4 and stored in an array. Using the brain material and the boron concentration array, twelve composite materials corresponding to 12 boron concentration levels (steps), were simulated using G4Element class. The pointers to each material were stored in an array of pointers to enable parametrisation of the geometry.

#### Voxelisation and material allocation

Allocation of a specific (heterogeneous) material composition to each individual voxel in Geant4, while also performing dose scoring, is a non-trivial task. In this work, in order to distribute corresponding boron concentrations to cells, the brain volume of 9 *cm* × 9 *cm* × 0.9 *cm* dimensions was divided into 20 *μm* side voxels, so that each voxel represented a cell. The divisions have been made in three layers. Firstly, the brain volume was divided in x and y direction to 3 × 3 × 9 *mm* ^3^ voxels (using *G4Replica*). As each replicated volume is indistinguishable from each other, another method, called nested parametrisation, was implemented for the second-layer division (9 mm to be divided by 3 to give 3 mm voxel size in z direction) as well as material parametrisation. The material for each voxel was selected from the array of materials based on replica numbers of each voxel in order to relate to the voxel s corresponding material (Using *G4NestedParameterization*). Finally, the central slice was divided into 20 *μm* side voxels to allow cell-based dosimetry. As the parametrised volumes have the limitation in that they cannot contain daughter volumes, this division was implemented using the so called Readout (RO) geometry method provided in Geant4. RO geometry is a parallel geometry which acts as a virtual tracking geometry, through which the real geometry is scanned. In RO geometry the scoring plane was divided to 20 × 20 × 20 *μm* ^3^ voxels (the scoring plane is the central plane in dark colour on the right side of Fig. 8). In other words, particles were tracked in the real geometry and spatial information of voxels (in terms of indices) in which interactions occurred and dose deposited were obtained from parallel geometry.

Given the potentially large amount of memory required for such a geometry, CPU time and RAM optimization methods were implemented: To parametrise the geometry to implement the heterogeneous material composition, the nested parametrisation method was utilized which requires much less memory for geometry optimization (compared to the commonly used direct three-dimensional parametrised volume method), resulting in faster navigation of systems with large number of voxels. Additionally, the brain volume was defined as a “sub-detector region”, using G4Region function, to allow for a more detailed simulation to occur only in this region. As a result, the processing speed increased markedly.

### Particle tracking and dose scoring

A BNCT beam model, simulating the epithermal spectrum from the LVR-15 reactor at Research Centre Rez^48^ with its realistic energy distribution was developed. The GBM phantom (geometry described above) was irradiated with this epithermal neutron beam model. Two beam sizes were considered: 2.5 and 3.0 cm radii corresponding to 2.0 and 2.5 cm radii CTV margin extensions, respectively. In the current simulation, the treatment was delivered in one fraction with a mean maximum tumour dose of 73.4 RBE -Gy, as per clinical protocols reported in literature^16, 42, 49, 50^. The mean maximum tumour dose in this study was considered as the dose averaged over the cells located in the GTV.

#### Dose conventions

Epithermal neutron beams designed for BNCT, predominantly produced by fission reactors and accelerators, have broad spectra and contain unwanted fast neutron and gamma contaminations. Four major dose components should be considered when such spectra interact with biological medium (the terminology has been adopted from Harvard-MIT BNCT treatment planning protocol^51^):

1. Boron dose from thermal neutron capture through ^10^B(n,*α*)^7^Li reaction (which is the sum of doses deposited by ^7^Li and alpha particles);
2. Thermal neutron dose as a result of thermal neutron capture by nitrogen atoms in biological tissue: ^14^N(n,p)^14^C;
3. Gamma dose from neutron capture by hydrogen atoms and also from gamma contamination in the incident neutron beam;
4. Fast neutron dose which is due to elastic and inelastic neutron scattering (e.g. ^1^H(n, n′)^2^H) and several possible nuclear reactions (e.g. ^16^O(n, *α*)^13^C)^30^.

In the current work, gamma contamination in the incident neutron beam was not taken into account and the gamma dose was produced from neutron capture by hydrogen atoms only.

In order to account for the variability of radiobiological effects of different physical dose components of a neutron beam spectrum incident on biological tissue, current clinical BNCT convention was adopted from IAEA-TECDOC-1223 BNCT treatment planning protocol^52^: each physical dose component (apart from the boron dose) was weighted by its respective Relative Radiobiological Effectiveness (RBE) factor, as listed in Table 5 ^11^, [Chapter 24]. The boron dose correction factor used is more complex as it depends on the boron carrier used to put boron in cells and is called Compound Biological Effectiveness (CBE).

**Table 5:** Relative biological effectiveness factors and CBE factor (only for boron dose) for four principal dose components^11^.

	RBE			CBE
	Photon *w*_*γ*_	Fast neutron *w*_*F*_	Thermal neutron *w*_*T*_	Boron *w*_*B*_
Brain tissue	0.5	3.2	3.2	1.3
Tumour tissue	0.5	3.2	3.2	3.8

Therefore, the total RBE-Gy dose, *d*_*wij*_, was calculated as:

4 $${d}_{wij}={w}_{\gamma }{d}_{\gamma ij}+{w}_{F}{d}_{Fij}+{w}_{T}{d}_{Tij}+{w}_{B}{d}_{Bij}$$

where *d*_*γij*_, *d*_*Fij*_, *d*_*Tij*_, and *d*_*Bij*_ were gamma dose, fast neutron dose, thermal neutron dose, and boron dose (i.e. sum of lithium and alpha doses) in voxel/cell ij, respectively.

For particle tracking, interaction processes and cross-sections determination, Geant4 packaged physics list, Quark-Gluon String Precompound Binary Cascade High Precision Neutron (QGSP_BIC_HP) was used. The High Precision data-driven neutron model (HP), included in the package, is used to track neutrons from 20 MeV down to thermal energies. Low energy parametrised neutron models are used when the data cannot be found in the HP database for any element. For neutrons with energies below a few eV, thermal neutron scattering from chemically-bound atoms is considered using thermal elastic scattering matrix S(*α*,*β*) tables (i.e. taking into account temperature-dependent momentum (*α*) and energy (*β*) transfer effects in crystalline structure of molecules. These effects occur at low energy neutrons^53^). In this packaged physics list, the standard EM model is adopted to model electromagnetic processes. Particles were assigned appropriate processes and cross sections along their trajectories. The tracks of ^7^Li and alpha particles from the boron capture reaction, gammas resulting from the neutron capture reaction, and all other particles and nuclear fragments from fast neutron reactions and thermal neutron capture were individually tracked in the simulation. The production threshold/cut off-values were set to 4 *μ*m for all particles other than gammas and to 0.01 mm for gamma particles. To ensure the correct level of precision, a step limit should be defined. In Geant4 a step contains information of a particle track which is updated upon the end of each step. A particle’s track is deleted if the particle disappears as a result of a decay or an inelastic scattering process (as is the case for fission and nuclear reactions). Therefore, a maximum step size, assumed to be sufficiently small with respect to the size of the sensitive volume, should be defined to calculate the energy deposition. In this simulation, the maximum step size was set to 4 *μ*m, corresponding to the minimum range of the alphas and ^7^Li nuclei. These production thresholds were assigned only to the brain volume (Fig. 8). Default thresholds (0.7 mm) were used for the rest of geometry.

Several techniques are provided in Geant4 to retrieve information (e.g. absorbed dose) through particle tracking. In this simulation a sensitive detector technique was implemented to score the dose from individual particles in the scoring plane.

Parallel simulations on 96-CPU 64-bit Linux clusters (South Australia (SA) Tizard supercomputer, the University of Adelaide) were performed for a total number of 5.8 × 10^9^ particles, in four simulation runs, each running for ∼17 days. Four dose matrices containing doses deposited by ^7^Li, alpha particles, gamma rays and neutrons (called the residual dose) were calculated for each core and the results were combined. The fast and thermal neutron doses were scored in a single matrix. This is possible as the RBE for both groups is the same (Table 5).

### Cell survival probability calculation

The four dose matrices calculated in Geant4 were exported into MATLAB. Using equation (4), the total RBE-Gy dose was calculated and was combined with the circular MEP model to obtain the cell survival probability for each cell using equation (5).

5 $$S{P}_{ij}=ME{P}_{ij}{e}^{-(\alpha {d}_{ij}+\beta {d}_{ij}^{2})}$$

where *MEP*_*ij*_ is the probability that the cell ij is a tumour clonogen, and *d*_*ij*_ is the absorbed dose in the cell ij. *SP*_*ij*_ denotes the survival probability of the cell ij. In the current work, the model consisted of a homogeneous population of GBM cells in terms of gene type and therefore uniform radiosensitivity was assumed with *α* = 0.281 *Gy* ^−1^ and *β* = 0.02 *Gy* ^−2^. These values are the means of Gaussian distribution of *α* and *β* values for several GBM cell lines reported in the previous work^35^.

In order to model tumour oxygenation levels (i.e. hypoxia), the Oxygen Enhancement Ratio (OER) was used previously for x-ray radiotherapy simulation. In case of BNCT, OER value of 1 was considered, as is the norm for high LET particulate radiation^25^.

Equations (6) and (7) were then used to calculate the number of surviving cells and SF for regions of interest:

6 $${\rm{surviving}}\,{\rm{cells}}\,{\rm{in}}\,{\rm{the}}\, \mbox{``} {\rm{region}}\mbox{''}=\sum _{i,j}S{P}_{ij}(i,j\in  \mbox{``} region\mbox{''})$$

7 $${S}{{F}}_{r{\rm{e}}{\rm{g}}{\rm{i}}{\rm{o}}{\rm{n}}}=\frac{{\rm{s}}{\rm{u}}{\rm{r}}{\rm{v}}{\rm{i}}{\rm{v}}{\rm{i}}{\rm{n}}{\rm{g}}\,{\rm{c}}{\rm{e}}{\rm{l}}{\rm{l}}{\rm{s}}\,{\rm{i}}{\rm{n}}\,{\rm{t}}{\rm{h}}{\rm{e}}\, \mbox{``} {\rm{r}}{\rm{e}}{\rm{g}}{\rm{i}}{\rm{o}}{\rm{n}}\mbox{''}}{{\rm{t}}{\rm{o}}{\rm{t}}{\rm{a}}{\rm{l}}\,{\rm{n}}{\rm{u}}{\rm{m}}{\rm{b}}{\rm{e}}{\rm{r}}\,{\rm{o}}{\rm{f}}\,{\rm{t}}{\rm{u}}{\rm{m}}{\rm{o}}{\rm{u}}{\rm{r}}\,{\rm{c}}{\rm{e}}{\rm{l}}{\rm{l}}{\rm{s}}\,{\rm{b}}{\rm{e}}{\rm{f}}{\rm{o}}{\rm{r}}{\rm{e}}\,{\rm{t}}{\rm{r}}{\rm{e}}{\rm{a}}{\rm{t}}{\rm{m}}{\rm{e}}{\rm{n}}{\rm{t}}}$$

SFs were evaluated in three regions: 1) within the beam region, i.e. the PTV; 2) within the penumbra region, defined in this study as the region extending 5.0 mm beyond the PTV; and 3) the total SFs, including in-beam, penumbra, and out of field (<1% dose coverage) regions. SFs were first calculated/predicted for a 2.0 cm CTV margin (equation (8)). The calculations were repeated four times using the dose matrices from four simulation runs.

The calculated SFs were compared with those calculated for homogeneous-hypoxic GBM tumour model for x-ray therapy published previously^34^.

8 $$\begin{array}{rcl}S{F}_{withinthebeam} & = & 100\times \frac{{\rm{surviving}}\,{\rm{cells}}\,{\rm{within}}\,{\rm{the}}\,{\rm{PTV}}}{{\rm{total}}\,{\rm{number}}\,{\rm{of}}\,{\rm{tumour}}\,{\rm{cells}}\,{\rm{before}}\,{\rm{treatment}}}\\ S{F}_{penumbraregion} & = & 100\times \frac{{\rm{surviving}}\,{\rm{cells}}\,{\rm{in}}\,{\rm{the}}\,{\rm{penumbra}}\,{\rm{region}}}{{\rm{total}}\,{\rm{number}}\,{\rm{of}}\,{\rm{tumour}}\,{\rm{cells}}\,{\rm{before}}\,{\rm{treatment}}}\\ S{F}_{total} & = & 100\times \frac{{\rm{surviving}}\,{\rm{cells}}\,{\rm{within}}\,{\rm{the}}\,\mathrm{PTV},\,{\rm{penumbra}}\,\mathrm{region},\,{\rm{and}}\,\mathrm{out} \mbox{-} \mathrm{of} \mbox{-} \mathrm{field}}{{\rm{total}}\,{\rm{number}}\,{\rm{of}}\,{\rm{tumour}}\,{\rm{cells}}\,{\rm{before}}\,{\rm{treatment}}}\end{array}$$

To further examine the pattern of SF within the beam and beyond, the differential SF was calculated in 0.5 mm steps and plotted versus distance from the tumour centre for the 2.0 cm CTV margin. The differential SF was defined as the ratio of the number of surviving tumour clonogens in a 0.5 mm wide rim at each distance from the tumour centre to the initial (total) number of tumour cells before treatment. A comparison of these results was made with differential SFs for x-ray therapy.

To obtain a quantitative measure of SF reduction with increased treatment margins, the CTV margin was extended by 0.5 cm to 2.5 cm and the beam size was increased to 3.0 cm (the radii of the GTV and the PTV margin remained the same). The change in SFs, as a result of the CTV increase by 0.5 cm, was calculated using equation (9).

9 $$S{F}_{change}=100\times \frac{S{F}_{2.0cm}-S{F}_{2.5cm}}{S{F}_{2.0cm}}$$
